# Carbon Microfiber-Doped Smart Concrete Sensors for Strain Monitoring in Reinforced Concrete Structures: An Experimental Study at Various Scales

**DOI:** 10.3390/s22166083

**Published:** 2022-08-15

**Authors:** Antonella D’Alessandro, Hasan Borke Birgin, Filippo Ubertini

**Affiliations:** Department of Civil and Environmental Engineering, University of Perugia, Via Goffredo Duranti 93, 06125 Perugia, Italy

**Keywords:** carbon-based fillers, concrete, carbon microfibers, multifunctional smart materials, structural health monitoring, dynamic monitoring, full-scale self-sensing elements

## Abstract

Concrete constructions need widespread monitoring for the control of their state of integrity during their service life. In particular, after critical events such as earthquakes, this type of structure may experience the formation and development of cracks and damage. A quick and affordable assessment of structural behavior is indicated to identify conditions of danger for users and the incipient collapse of structural elements. This work presents investigations on multifunctional concretes with self-sensing capabilities to carry out static and dynamic monitoring. The materials were produced by the addition of conductive carbon microfibers to the concrete matrix. Electrical and sensing tests were carried out on samples with small-, medium-, and full-scale dimensions. The tests demonstrated the good electrical and electromechanical properties of the proposed smart concrete sensors, which appear promising for their use in civil elements or structures. In particular, tests on real-scale beams demonstrated the capability of the material to monitor the dynamic behavior of full-scale structural elements.

## 1. Introduction

Being a very old material, concrete has been the most used construction material in civil engineering for many years, and its composition is still evolving [1]. It is a composite material made of cement and aggregates, and is durable under high compression loads. The applications of concrete in civil structures and infrastructure is in its reinforced form, in which concrete and stainless-steel reinforcing bars work to bear structural loads in terms of compression, shear forces, and bending moments. Compared to the service life of steel structures, reinforced concrete designs have shorter service life expectancy, conventionally considered about 50 years [2]. In Europe, around 35% percent of residential structures are older than 50 years, indicating that there exists an unquestionable need for assessment and maintenance of those buildings [3]. This requirement can be achieved by use of structural health monitoring (SHM) systems, which consist of systematic inspection, identification, and assessments of civil structures and infrastructures [4].

In the last years, SHM has evolved and advanced rapidly [5]. Improvements have occurred in the sensor technologies [6,7], as well as in algorithm development [8,9], increasing the effectiveness of processing the collected signals. As a matter of fact, the design of an SHM system is an optimization between the types and numerosity of samples, and the structure of the monitoring system [10,11,12,13]. A possible solution could be the choice of a sensor responsible for each degree of freedom of the structure, but this is far from ideal because it is unsustainable in terms of costs and operational maintenance due to the fragility of the sensors. The optima; practice of SHM involves the identification of critical structural points, the appropriate energy source for the sensors and the system, the effective application of the sensors to ensure a good quality of bonding, and finally the reliable transmission and processing of the collected data. The more common traditional sensors for SHM are: (i) inertial sensors, such as accelerometers and gyroscopes [14]; (ii) displacement/deformation-based sensors [15], such as GPS for large scale deformations [16], FBGs, and strain gauges for the local structural members; and (iii) external sensors, such as high speed and high resolution cameras and velocimeters [17]. Currently, SHM applications involve small structures or limited structural parts due to system complexities and financial costs. Availability in the market of simple and inexpensive SHM systems capable of making accurate monitoring could represent great progress for the control and maintenance of structures and infrastructure during service life [18] and the identification of incipient damages or collapses that could be dangerous for users. A possible solution to achieving widespread structural monitoring systems could be the use of multifunctional self-sensing composites. Such materials are capable of estimating their strain states under external loads and displacements, and are able to identify variations in strain/stress fields. Moreover, they offer longer operational durability and excellent structural bonding because they can be embedded in the structures or can constitute whole structural elements [19,20]. In this way, the structure itself could sense its state of strain and recognize variations in its behavior and integrity. The self-monitoring activity is achieved through the correlation of changes in strains/stresses and changes in electrical properties. For this reason, the building material should be electrically conductive, a property than can be obtained with the addition of conductive fillers to its matrix [21,22]. Typically, a voltage is applied on conductive electrodes connected to the material and the variation in electrical current or voltage is measured. The applied signal can be DC, AC, or biphasic [23,24]. The sensitivity of the obtained smart construction composite depends on the piezoresistance of the fillers, the electrical properties of the matrix, and the contact resistance between the fillers and the matrix and between the composite and the electrodes [25,26]. Suitable fillers for these types of applications are steel-based or carbon-based [27,28,29,30,31]. Examples of possible suitable additions are carbon nano- and micro-fibers, carbon nanotubes, graphene, graphene nanoplatelets, graphite, and carbon black [32,33]. The presence of the fillers can enhance both the electrical and mechanical properties of the materials [34,35]. Among all the building materials, concrete and cement-based materials in general appear particularly promising for smart self-sensing applications. This is due to the composite nature of such materials, which permits the addition of inclusions of various dimensions, even at the nano-scale [36,37]. However, cementitious composites are water-based; thus, the addition of hydrophobic fillers such as those mentioned above may be difficult. The proper dispersion of the fillers, which guarantees sufficient homogeneity and isotropy of the material, can be achieved through the use of dispersants, mechanical stirrers, sonication, or functionalization of the components [38,39]. The choice of the optimal dispersant procedure is delicate and depends on the specific application of the composite, because the presence of a surfactant or the use of different mixing procedures can affect or modify the enhanced properties of the fillers and thus the properties of the final mix. From literature, sonication shows better dispersion effectiveness for carbon fillers, especially for nano-sized ones. However, such a method is suitable for small amounts of materials and cannot be simply applied to larger-scale applications such as structures or infrastructure in civil engineering. Micro-sized inclusions could solve such a scalability issue because they can be mechanically added to cementitious materials, as with the other components. Literature about self-sensing cement-based materials are generally oriented towards the investigation of small samples or rheological aspects [39,40]. In-depth research about full-scale application of smart concretes has not yet been carried out. The authors of this paper are developing a research campaign about self-sensing cement-based samples and elements for monitoring of concrete structures [36,41], towards the realization of full-scale smart concrete elements. Starting from the investigation of the electrical and sensitive properties of small concrete samples, this paper is aimed at investigating the monitoring capabilities of medium- and real-scale elements. Section 2 and Section 3 describe the components’ characteristics and the experimental setup and procedures. Section 4 indicates the theoretical behavior of the investigated smart concretes. Section 5 and Section 6 report and comment on the experimental tests on small-, medium-, and full-scale samples. Section 7 concludes the paper.

## 2. Materials and Samples

This research is a multi-scale investigation of different types of samples made of smart concrete. The components, the electrical setup, and the test instrumentation have been tailored and optimized for the different applications.

### 2.1. Components

The sample base material was a concrete designed for a reference characteristic resistance of 30 MPa. The cement was Portland 42.5R; the fine and coarse aggregates were taken from a quarry. The mix design is reported in Table 1. To obtain the smart concrete, the construction material was doped with carbon microfibers (CMF) type Sigrafil provided by SGL Carbon, which were added at different percentages with respect to the weight of the cement. The physical and mechanical properties of the fillers are reported in Table 2.

The aggregates adopted for the small- and real-scale samples possessed tailored characteristics due to the dimensions of the element. The fillers were added to the concrete and then mechanically dispersed in order to obtain a homogeneous material. The quality of the dispersion was examined through microscope investigations at different magnifications using an optical microscope and a scanning emission microscope.

**Figure 1:**
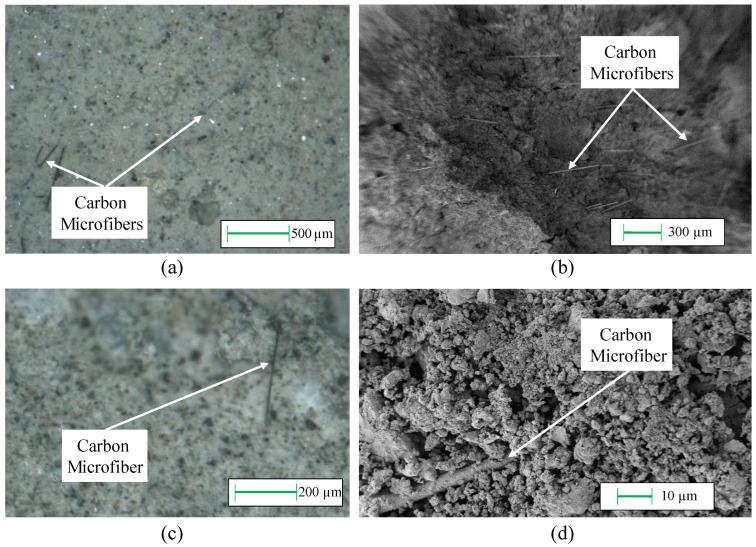
Micrographs of a hardened smart concrete fragment using (**a**,**c**) an optical microscope and (**b**,**d**) SEM at different magnifications.

**Figure 2:**
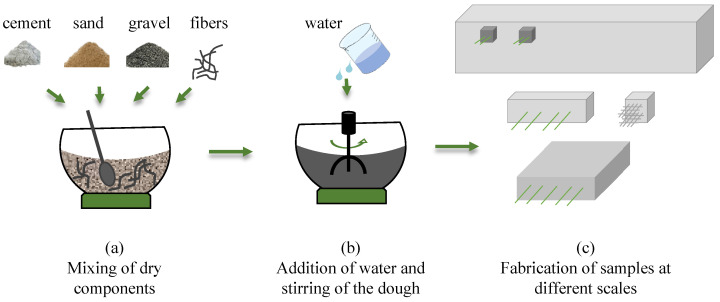
Preparation procedure for smart concrete.

Figure 1 reports the micrographs of a hardened fragment of concrete doped with carbon microfibers, where the presence and the good dispersion of the fillers are clearly visible. Pictures at lower magnifications (Figure 1a,b) show the absence of agglomerations and the distance between the single fibers, while scans at higher magnifications (Figure 1c,d) demonstrate the integrity of the single separated fibers. Figure 2 shows the preparation procedure adopted for all the samples. The first part of the process, up to the production of the fresh concrete by mixing of cement, aggregates, fibers, and water, is similar (Figure 2a,b). After the mix of the components, the dough was poured into oiled plastic and steel molds in the case of small cubes, small beams, and plates; and wood-made molds for real-scale beams. The wood molds were prepared with the designed dimensions and the reinforcement bars were placed before pouring the fresh concrete.

The small and medium samples were cured at laboratory conditions, while the full-scale beams were cured at external environmental conditions. The curing time was at least 28 days.

### 2.2. Small-Scale Samples

**Figure 3:**
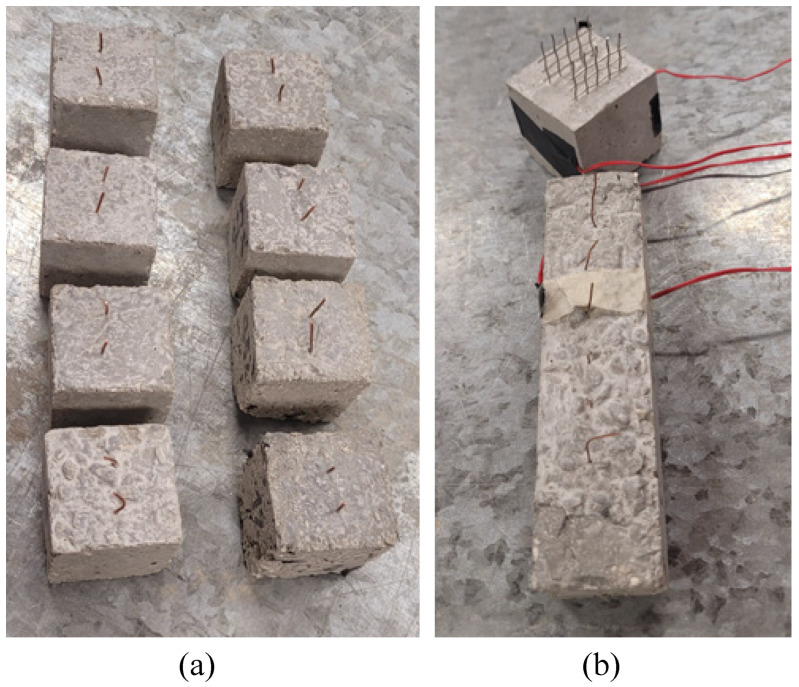
Small-scale samples: (**a**) cubes with 4 cm sides doped with increasing percentages of CMF; (**b**) cube with 5 cm sides and small beam with 0.05% of CMF.

The small cubes used for the investigation of the monitoring capabilities of the smart concrete at increasing doping levels had sides of 4 cm. The carbon fillers were added at the percentages of 0.01, 0.025, 0.05, 0.1, 0.25, 0.5, and 1% with respect to the weight of the cement. Before the solidification of the material, two copper wires with a diameter of 0.8 mm were embedded in the central part of each cube at a mutual distance of 2 cm as electrodes for the electrical measurements. Figure 3a shows the complete series of the small cubes. From the electrical and electrical tests performed on them, the percolation threshold of the conductive net within the matrix and the sensitivity of the doped composites, were analyzed.

A cube sample with sides of 5 cm was then prepared for in depth investigation of the sensing capabilities of the smart concrete. This sample was doped with 0.05% of CMF. For this type of sensor, stainless steel nets were adopted as embedded electrodes in order to evaluate the influence of the type and configuration of the conductive electrodes on the sensitivity of the material. Furthermore, small beams were prepared, with dimensions of 160 × 40 × 40 mm${}^{3}$ as the standard samples for mortars. Copper wires with diameter of 0.8 mm were embedded in the central line along the greater dimension. Figure 3b presents the photographs of the small cube and the small beam samples. For the cube and small beam samples, the placement of the electrodes was designed so as to be along the direction of the applied loads in the electromechanical tests.

### 2.3. Middle-Scale Samples

**Figure 4:**
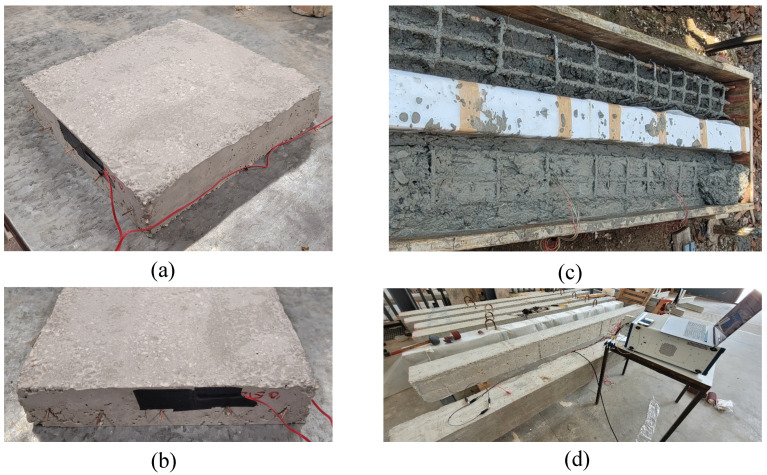
Medium- and full-scale samples: (**a**,**b**) plate with linear embedded electrodes; (**c**) beams during preparation; (**d**) beams after the curing period.

The middle-scale plate was produced with concrete doped with 0.05% of CMFs (Figure 4a). Its base had dimensions of 250 × 250 mm${}^{2}$, while its thickness was 50 mm. The electrodes—0.8 mm copper wires—were placed in parallel on the plane at the median thickness of the plate and at a mutual distance of 50 mm.

### 2.4. Full-Scale Samples

**Figure 5:**
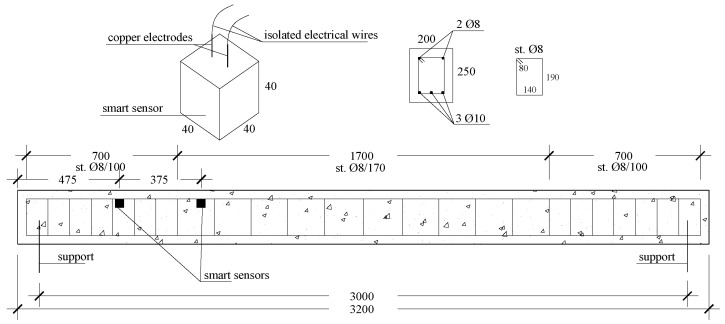
Sketch of the dimensions and reinforcements of the full-scale beam with embedded smart sensors, cross-section, and representation of a single smart sensor.

The full-scale beam had a cross-section of 200 × 250 mm${}^{2}$ and a length of 3200 mm, as depicted in Figure 5. The element was supported by two embedded steel profiles placed at a distance of 3000 mm. The beam reinforcement was constituted by two longitudinal ϕ8 rebars, and three ϕ10 rebars on the extrados and intrados, respectively. The ϕ8 stirrups were placed at a distance of 170 mm in the central part of the beam and at a distance of 100 mm in the lateral edges. The smart cubic samples were embedded in the internal concrete core at the extrados side at distances of 475 mm and 850 mm from the support. These were cubes with 40 mm sides instrumented with two 8 mm copper wires and two lateral strain gauges. All the cables were insulated and connected to the outside by use of further insulated cables. All the samples were covered by insulating tape. The mutual distance between the smart sensors was 375 mm.

## 3. Setups and Instrumentation

**Figure 6:**
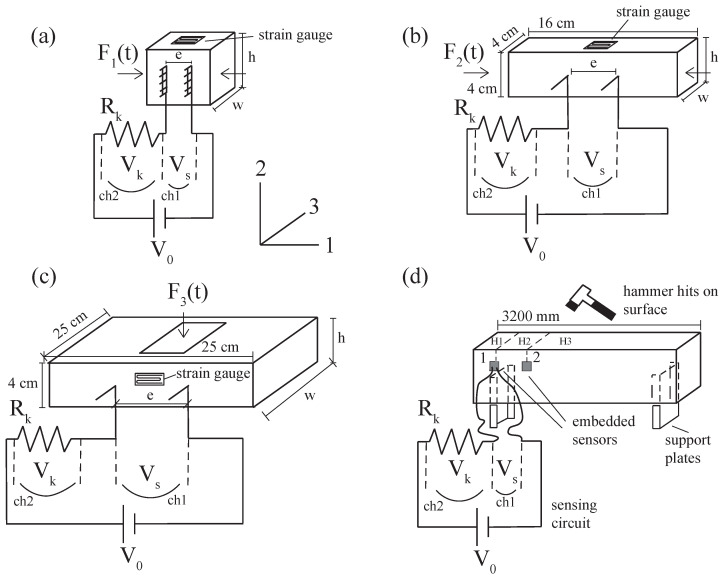
The setups and related electrical circuits: (**a**) the typical cube sample; (**b**) the beam sample; (**c**) the plate sample; and (**d**) the full-scale beam with embedded smart concrete sensors plotted together with cross-section.

The setups used in this study are illustrated in Figure 6, which describes each type of test specimen and load applications. The various test setups were configured to investigate the strain sensing capabilities of samples with varying geometries and electrode designs for embedded applications. The goal was to investigate the flexibility of such sensors for different structural elements or load applications.

During the electromechanical tests, the sample resistances were calculated through the use of a shunt resistor of known value R${}_{k}$. In the test configurations, the tested sample and the shunt resistor were connected in series. The input voltage indicated by V${}_{0}$ was a 20-volt peek-to-peek square-wave with frequency of 1 Hz. The input of the biphasic square-wave was adopted to reduce the polarization effect, which creates an upwards drift of the resistance of cement-based materials over time and typically occurs with DC inputs. Only V${}_{0}$ represented in Figure 6d was 10 V DC, which is suitable for dynamic tests on the full-scale beams. V${}_{s}$(t) and V${}_{k}$(t) are potential difference time histories acquired through the tested sample and shunt resistor, respectively. The data rate for voltage acquisitions was 10 Hz for the cases in Figure 6a–c, which allowed the selection of an 80% charge point for each positive part of the square-wave to build the resistance time histories. The data rate of the configuration in Figure 6d was 1000 Hz. The voltage time histories were recorded; then, the electrical resistance time history of the inspected specimen, R(t), was calculated using Ohm’s law, as reported in Equation (1), assuming that the electrical current was one directional inside the specimen.

(1) $$R\left(t\right)=R_{k}\frac{V_{s}\left(t\right)}{V_{k}\left(t\right)}$$

The loads F${}_{1}$(t) and F${}_{2}$(t), represented in Figure 6a,b, were compression loads applied along the direction of the electrodes (labeled as direction 1). The load time history was constituted by 10 triangular cycles with peak values of 9 kN and a load increase rate of 2 kN/s. F${}_{3}$(t) reported in Figure 6c was applied over the surface of the plate sample. In this case, the load time history was formed by 4 triangular cycles with peak values of 9 kN and a load increase rate of 2 kN/s. The load for the tests of Figure 6d was constituted by hammer hits placed on three different locations on the beam, indicated by H1, H2, and H3.

During the tests, the voltage input V${}_{0}$ was sourced from a NI-PXIe 4138, while V${}_{s}$ and V${}_{k}$ were recorded through the 24-bit analog-to-digital converter NI-PXIe 4302. The cube, beam, and plate samples were instrumented by 2-mm monoaxial strain gauges, as illustrated in Figure 6, and the strains were measured by an NI-PXIe 4330. All the acquisitions were performed under the LABVIEW environment. The mechanical load time histories F${}_{1}$(t), F${}_{2}$(t), and F${}_{3}$(t) were applied by a dynamic compression machine type IPC Global UTM14P, capable of applying a maximum load of 14 kN. For the signal processing, the frequency analysis was conducted using the fast Fourier transform and wavelet transform libraries of MATLAB.

## 4. Electrical and Dynamic Modeling

### 4.1. Piezoresistivity of the Samples

The sensitivity of the smart concrete sensors investigated in this study depended on the piezoresistivity of the composite and its electrical setup, which produced electrical signals correlating with the varying strain-states of the sensor. The sensors were assumed to be rectangular solid elements; thus, the resistance of any segment between electrodes could be formulated as follows:

(2) $$R=\rho_{r}\frac{e}{A}$$

where $\rho_{r}$ is the resistivity of the material, *e* is the distance between electrodes, and *A* is the cross-sectional area between the electrodes. Using the notation of Figure 6, Equation (2) can be rewritten as:

(3) $$R=\rho_{r}\frac{e}{h\cdot w}$$

where *e* is the distance and h·w is the rectangular cross-sectional area of the segment between electrodes. Considering the setups introduced in Figure 6a,b, the fractional variation of the resistance caused by the applied loads can be formulated assuming the material as isotropic and the load applied along axis 2 using Poisson’s ratio, ν, and conversion $\varepsilon_{2}=\varepsilon_{3}=-\nu \varepsilon_{1}$, where ε denotes strains along the axis direction associated with the subscript, indicated in Figure 6. For the plate setup introduced in Figure 6c, the strain conversion results are $\varepsilon_{1}=\varepsilon_{3}=-\nu \varepsilon_{2}$, due to the direction of the applied load. From the previous equation, the normalized resistance variation can be obtained:

(4) $$\frac{dR}{R}=\frac{d\rho_{r}}{\rho_{r}}+\frac{de}{e}-\frac{dh}{h}-\frac{dw}{w}$$

After the substitution of the terms for the cube and small-sized beam sensor element, the following equation can be determined:

(5) $$\begin{array}{cc} \frac{dR}{R} & =\frac{d\rho_{r}}{\rho_{r}}+\varepsilon_{1}-\varepsilon_{2}-\varepsilon_{3} \end{array}$$

(6) $$\begin{array}{cc} \frac{dR}{R} & =\frac{d\rho_{r}}{\rho_{r}}+\left(1+2\nu \right)\varepsilon_{1} \end{array}$$

while for the plate sensor, the following can be obtained:

(7) $$\frac{dR}{R}=\frac{d\rho_{r}}{\rho_{r}}-\varepsilon_{2}$$

Equations (6) and (7) yield to Equations (8) and (9), which represent the piezoresistivity models for the cube and small beam, and the plate sample, respectively:

(8) $$\begin{array}{cc} \lambda =\frac{\frac{dR}{R}}{\varepsilon_{1}} & =\frac{\frac{d\rho_{r}}{\rho_{r}}}{\varepsilon_{1}}+\left(1+2\nu \right) \end{array}$$

(9) $$\begin{array}{cc} \lambda =-\frac{\frac{dR}{R}}{\varepsilon_{2}} & =-\frac{\frac{d\rho_{r}}{\rho_{r}}}{\varepsilon_{2}}+1 \end{array}$$

where λ is the gauge factor, which characterizes the sensing capability of the smart material. The gauge factor aims to correlate the variation of resistance to the induced strain for a smart concrete sensor. In the developed formulations of gauge factor, the term $d\rho /\rho /\varepsilon_{2}$ represents the sensitivity due to the piezoresistive effect, while the other term indicates variations due to body deformations of the material.

### 4.2. Natural Frequency of the Beam

In the experimental tests, the dynamic characteristics of a real-scale beam equipped with embedded smart concrete cube sensors were evaluated. Analytical characterization of the beam for the dynamical properties was carried out to verify and compare the results attained through the smart sensors.

The natural first mode vibration frequency of the beam was calculated by adopting the Euler–Bernoulli beam theory. The boundaries of the beam were assumed to be clamped due to the profile used for the supports of the beam. The exact solution for the first bending mode is shown in Equation (10).

(10) $$2\pi f_{1}=\frac{4.73^{2}}{L^{2}}\sqrt{\frac{EI}{\rho_{m}A_{b}}}$$

Inside Equation (10), $f_{1}$ is the first bending mode frequency (Hz), *L* is the span between supports, equal to 3 m, and *I* is the moment of inertia of the composite reinforced concrete section. Considering a cover thickness of 25 mm according to the design of the cross-section, the neutral axis occurred at a distance of 127 mm from the top surface. Based on the experimental setup, *I* was calculated as $2.71\times 10^{-4}\;m^{4}$. $\rho_{m}$, the density of the unloaded beam was 2500 kg/m${}^{3}$; $A_{b}$ was the cross-sectional area of the beam, and 4.73 was the boundary value for a beam with clamped ends. Finally, Young’s modulus of the concrete, *E*, had a value of 22 GPa. With these given parameters, the natural frequency was found to be 88 Hz.

## 5. Experimental Results

### 5.1. Small Cube Samples with Varying CMF Concentrations

**Figure 7:**
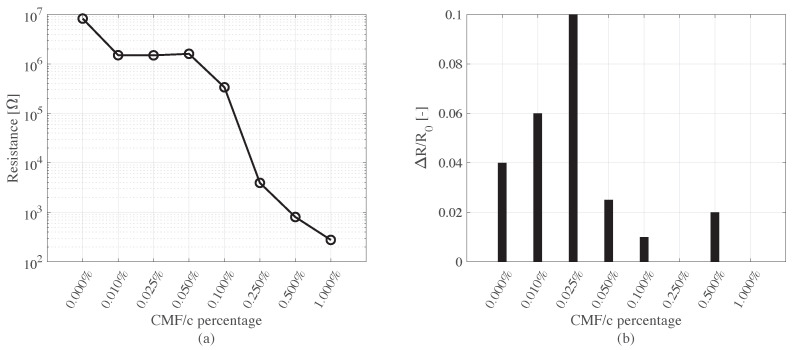
The results of the percolation study (**a**) and sensitivity tests (**b**) done on small smart concrete cube samples.

The results of electromechanical tests done on the small cube samples are summarized in Figure 7. According to the resistance evolution with increasing CMF percentage (Figure 7a), the percentage of 0.05%w CMF/c represents the level beyond which the resistance of the samples dropped by orders of magnitude. It is worth noting that the percolation curve is in fact affected by some statistical dispersion, which is the result of variability in microfiber distribution. Evaluating such a statistical variability is not in the aims of the present paper, where one sample per fiber amount is considered; however, the order of magnitude difference observed between the resistance values in the transition zone beyond 0.05% demonstrated the percolation of the carbon microfibers. The CMF concentration of 0.05%w with respect to the cement weight was also consistent with a previous study that inspected similar materials [42]. The best performing composite occurred at the percolation; however, as mentioned already, the presence of an imperfect distribution of the inclusions could determine variability in the electrical behavior and in the gauge factor as well. Again, investigating the statistical distribution of the gauge factor goes beyond the purposes of the present study. Figure 7b shows the fractional variations of sample resistances during the electromechanical compression tests using step increasing loads, with a maximum value of 7 kN. The results show increased sensitivity up to a CMF/c percentage of 0.025%. The trends followed by the values in Figure 7b are in accordance with the electrical percolation of the conductive fillers. Based on these two results, 0.05% appears to be a more suitable doping level than 0.025% for sensing applications on full-scale elements, especially concerning the scalability issues that may arise for the sensors with larger dimensions. The sensitivity of the selected composite design has been further investigated.

### 5.2. Strain Sensing Capabilities of Cube and Beam Samples under Compression Loads

**Figure 8:**
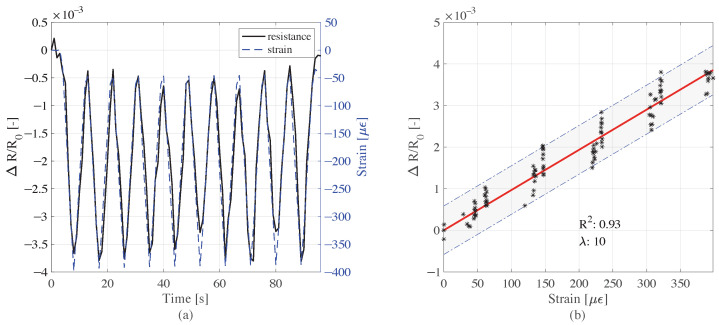
Sensitivity response of the cube sample under the cyclic compression load: (**a**) variation of resistance and the induced strain time histories; (**b**) resistance variation versus strain, and linear model of sensing.

**Figure 9:**
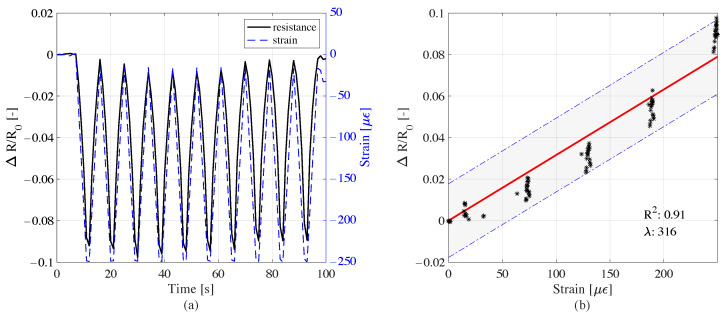
Sensitivity response of the beam sample under the cyclic compression load: (**a**) variation of resistance and the induced strain time histories; (**b**) resistance variation versus strain, and linear model of sensing.

The cube sensor with 5 cm sides and 0.05% CMF exhibited good strain sensing performance under cyclic load history. The variations in resistance and strain shown in Figure 8a appear well correlated, as verified by the linear model of sensing, with an R${}^{2}$ of 0.93, as shown in Figure 8b. The gauge factor for the cube sensor sample was 10 according to the linear model of sensing. Analyzing a different geometry, i.e., the small beam sensor element, the electromechanical tests produced results similar to those obtained from the cube sensor; the resistance time history followed the trend of the strain time history, as reported in Figure 9a.

The linear model of sensing for the beam element, shown in Figure 9b, exploits the high linearity between the variation of resistance and the induced strain by the applied compression loads. The gauge factor for the beam element , according to the linear model of sensing, was 316, significantly higher than that obtained from the cube sample.

### 5.3. Load Sensing Performance of Plate Sample under Surface Compression

**Figure 10:**
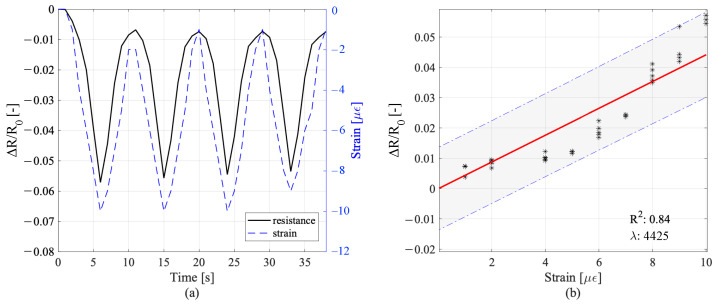
Sensitivity response of the plate sample under the cyclic surface loads: (**a**) variation of resistance and the measured strain time histories; (**b**) resistance variation versus strain, and linear model of sensing.

The plate sample was subjected to cyclic surface loads perpendicular to the electrode alignment. The variation in resistance and the strain time histories measured at the location of strain sensor according to the cyclic loading are plotted in Figure 10a, while the relative linear model is shown in Figure 10b. While the cube and beam sensors were tested under axial compression, the plate sensor was subjected to surface loads applied on the middle segment of the plate. The deformation that occurred around the strain gauge in this load configuration depended on two counteracting mechanisms: the expansion caused by Poisson’s effect and the compression due to the bending of the plate sample. The two effects were opposite, thus decreasing the strain value read at the location of the strain gauge. The strain time history for this experiment served as a reference to evaluate the linearity of the variation of resistance. The linear model exhibited good linearity and the gauge factor appeared to be very high; this last result is probably due to the position of the strain gauge on the plate sensor.

### 5.4. Dynamic Sensing Capabilities of Smart Concrete Sensors in Full-Scale Beam

**Figure 11:**
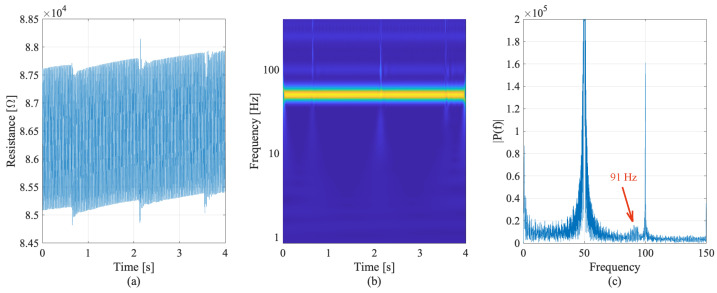
Resistance time history (**a**), frequency–time analysis (**b**) and frequency response (**c**) of first embedded smart concrete cube sensor during hammer hit tests.

**Figure 12:**
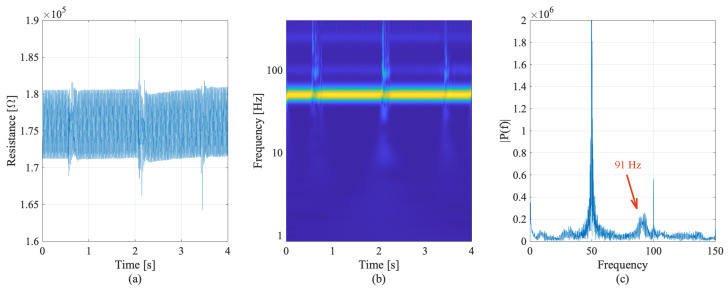
Resistance time history (**a**), frequency–time analysis (**b**), and frequency response (**c**) of second embedded smart concrete cube sensor during hammer hit tests.

The electrical response of the embedded cubes selected from the dynamic tests, and the related frequency analysis are shown in Figure 11 and Figure 12, for the first cube sensor and the second cube sensor, respectively (Figure 6d).

Accordingly, Figure 11a plots the resistance time history, Figure 11b plots the frequency–time analysis of the sensors, and Figure 11c plots the frequency response of the first embedded cube sample during the dynamic test with hammer hits. Inspecting the results coming from the first embedded sensor, the effect of hammer hits are visible in the resistance time history. However, the resistance time history exhibits a periodical noise that affects the quality of the readings. In the frequency–time plot, the permanent noise, probably due to the electrical current system, clearly determines the peaks at 50 Hz and multiples. However, the excitation of first bending mode is visible at a frequency of 91 Hz. The peak frequency obtained from the experimental test is very close to the first bending mode calculated through analytical analysis of the full-scaled beam. This frequency is verified by the outputs of the second dynamic tests carried out on the other embedded sensor.

The second sensor appeared to be more sensitive, producing better results in the dynamic analysis of the full-scale beam. This might be related to the location of the second sensor further from the support than the first embedded sensor. Inspecting the resistance time history in Figure 12a, the impulses created by hammer hits were more evident. The instrumentation noise was present as in the first experiment, but the frequency peak of the first mode at 91 Hz was more evident both in the frequency–time graph (Figure 12b) and frequency response of the signal (Figure 12c). The overlapping findings from separate tests highlight that the readings are reliable and repeatable. Such tests, as planned, represent evidence of the good performance of the smart material for sensing applications on real-scale structures.

## 6. Discussion

Smart concrete, as a strain-sensing multifunctional structural material, shows promising capabilities in terms of widespread SHM of the civil structures. This study inspected the strain-sensing capabilities of CMF-doped concrete with different sensor geometries, doping level, and electrical setups. Small-, medium- and full-scale elements made of smart concrete were tested. The study investigated two types of monitoring, namely sensing at low-varying loads through cyclic loads, and dynamic sensing aimed at detecting the dynamic characteristics of a real-scale beam through embedded sensors placed inside the element excited by hammer hits.

The study first investigated the strain sensing potential of smart concrete sensors with different geometries, including the sensing performance and the best amounts of conductive fillers to enhance the sensitivity of ordinary concrete. The results concerning strain-state sensing appeared quite promising; the linear models that correlated strain readings and electrical responses generated by the smart concrete sensors showed high reliability. Accordingly, the small cube and beam sensors appear useful to be employed as embedded strain sensors for SHM purposes, where such smart sensors can be placed on critical structural members to produce a long-lasting and feasible monitoring system.

The plate sample of this study had larger dimensions and was loaded on the middle segment of its top surface in order to simulate a bending load for horizontal concrete elements with self-sensing material. The tests demonstrated that signals were highly correlated to the cyclic loads. The plate sample was instrumented by a traditional strain gauge, and the sensing results demonstrated that such a tailored design of electrodes and element characteristics has advantages over traditional strain gauges because the smart sensor used a larger volume for sensing and, as a result, was less affected by limiting factors, such as the placement of the loads on the sensor, local surface conditions, and the local placement of the strain-sensors. In general, the sensors’ responses were found to be scaled to the induced strain and the response time of the material was observed to be short. Moreover, the residual drifts due to material deformations were negligible.

The dynamic monitoring tests with embedded concrete sensors generated significant results with important remarks to discuss. The study with full-scale elements demonstrated that dynamic analysis of the structural members and the detection of first modes is feasible with the use of smart multifunctional materials. However, further discussion is necessary to clarify design limits. Instrumentation noise with a constant frequency was present during the tests. Such noise was probably due to the current of the electrical system and the high impedance of the electrical sensing circuit, which affects the readings of the analog-to-digital converter of the data acquisition equipment. To address this issue, the electronic system design could be tailored to reduce the impedance of the sensing circuit, the hardware could be selected to be compatible to high impedance levels, and a post-processing filter could be developed accordingly. Moreover, such a limiting factor could be overcome enhancing the strain sensitivity of the smart material in order to generate signals with great amplitude that will not be affected by the noise. Therefore, the optimization of the multifunctional system design considering all the components is pivotal for the development of advanced applications in the field of engineering. Overall, the obtained results support the possibility of cost-efficient goal-oriented widespread structural health monitoring systems with smart concrete sensors suitable to be applied on structures and infrastructure. Possible applications could be continuous monitoring during the service life of constructions, or the quick assessment of the structural integrity and performance after critical events such as earthquakes.

## 7. Conclusions

This paper presents the first results of sensing tests carried out on samples made of smart concrete. The monitoring capability was obtained through the addition of carbon microfibers in the construction material. Small-, medium- and full-scale samples with different characteristics and setups were tested by electrical and electromechanical experiments, with various types of applied loads. The aim of the research was to analyze the potential of such a smart concrete to be utilized in real-scale structures. As a matter of fact, the presence of coarse aggregates and the steel rebars in reinforced concrete elements could constitute an issue for the sensing performance of the smart sensors. To this end, the study presents experimental results on full-scale elements towards the use of embedded smart concrete sensors in real constructions, and discusses the strengths and the limitations of such monitoring systems. The electrical and electromechanical tests on small samples with increasing amounts of conductive piezoresistive fibers demonstrated variations in sensitivity and permitted the identification of the most promising filler percentage for monitoring applications. The sensing tests on small samples and on the plate demonstrated the effectiveness of different types of electrical setups and load applications. Finally, the dynamic characterization tests on a full-scale reinforced concrete beam instrumented with embedded smart concrete samples demonstrated the promising sensing capabilities of such novel self-sensing sensors for applications in real structures and infrastructure. Based on the good results obtained by this work, future experimental campaigns can be planned and carried out to examine the statistical dispersion of electromechanical properties over a large number of sensors and to evaluate the performance of full-scale structural elements produced entirely or partially with smart sensing concrete and tailored electrical setups.

## Figures and Tables

**Table 1:** Mix design for smart concrete.

Components	kg/m${}^{2}$	CMF/c Percentage (%)	kg/m${}^{2}$
Cement	420	0.000	0.000
Aggregates	1830	0.010	0.042
Water	212	0.025	0.105
		0.050	0.210
		0.100	0.420
		0.250	1.050
		0.500	2.100
		1.000	4.200
w/c ratio	0.5		

**Table 2:** Properties of carbon micro fibers adopted in the experiments.

Property	Units	Values
Density	g/cm${}^{3}$	1.8
Fiber length chopped	mm	6
Filament diameter	μm	7
Tensile strength	GPa	4.0
Tensile modulus	GPa	240
Elongation at break	%	1.7
Single filament resistivity	μΩm	15
Bulk density	g/L	-
Sizing type	-	Glycerin
Compatible with	-	Water-based systems

## Data Availability

The data is available upon a reasonable request.
